# Carob Seeds: Food Waste or Source of Bioactive Compounds?

**DOI:** 10.3390/pharmaceutics12111090

**Published:** 2020-11-13

**Authors:** Debora Santonocito, Giuseppe Granata, Corrada Geraci, Annamaria Panico, Edy Angela Siciliano, Giuseppina Raciti, Carmelo Puglia

**Affiliations:** 1 Department of Drug Sciences, University of Catania, 95125 Catania, Italy; debora.santonocito@outlook.it (D.S.); panico@unict.it (A.P.); edysiciliano@hotmail.it (E.A.S.); racitigi@unict.it (G.R.); 2 Istituto Chimica Biomolecolare–C.N.R., Via Paolo Gaifami 18, 95126 Catania, Italy; giuseppe.granata@icb.cnr.it (G.G.); corrada.geraci@icb.cnr.it (C.G.)

**Keywords:** carob fruit, seed carob, food waste, ORAC, polyphenols

## Abstract

(1) Background: For centuries, carob fruit has been used in the food field, while carob seeds have been mainly considered as food waste. Nowadays, there has been considerable attention toward the recovery of the waste plant matrices as possible sources of functional compounds with health properties. Therefore, our goal was to evaluate the health properties of carob seed extracts, and to study the effects of the ripening process on the chemical composition of the extracts. (2) Methods: After the mechanical separation of seeds from carob fruit, an ultrasound-assisted extraction (UAE) was performed to maximize and preserve the quality of bioactive compounds. Seed extracts were characterized by high-performance liquid chromatography (HPLC) and liquid chromatography/mass spectrometry (LC/MS) for the content of bioactive polyphenols, and were finally analyzed by oxygen radical absorbance capacity (ORAC), NO Scavenger (NO) and advanced glyoxidation end products (AGEs) assays, in order to estimate the antioxidant potential of the active compounds. (3) Results: Although both seed extracts of carob unripe (CAR-UR) and ripe (CAR-R) showed an interesting antioxidant activity, CAR-R had greater activity due to the procyanidins content. (4) Conclusions: Based on the obtained results, carob seed extracts could be regarded as interesting source of bioactive antioxidant compounds for a potential application in nutraceutical and food supplement fields.

## 1. Introduction

Carob (*Ceratonia siliqua* L.) is an evergreen tree widely cultivated in the Mediterranean area. It belongs to the Leguminosae family and its scientific name derives from the Greek word “*kera*”, due to the keratomorphic shape of the fruit, and the Latin word “*siliqua*”, related to the hardness and shape of the pods [1,2]. The carob fruit (bean) is called pod and consists of two parts: the seeds (10%) and the pulp (90%). The pulp comprises a leathery outer layer (pericarp) and a softer inner region (mesocarp) where the seeds are found; meanwhile, the seeds are composed of three layers: shell, endosperm and embryo. Seeds are brown, hard, 10 mm long and with a weight of about 0.2 g per seed [3,4,5]. Due to the homogeneity in weight of the seeds, it is popularly believed that the carat, the unit of weight for gemstones, is derived from the carob seeds [6].

Carob pulp contains numerous bioactive compounds such as sugars, cyclitols, polyphenols, amino acids, fibers and minerals, while the composition of carob seeds includes gum, polyphenols and proteins [1]. Due to the chemical composition, carob exhibits a powerful antioxidant activity, and possesses many valuable therapeutic functions, such as lipid-lowering [7], anti-cardiovascular, anti-proliferative [8,9] and nephroprotective properties [10]. Furthermore, during the ripening of the fruit, many biochemical reactions occur with consequent modifications of the final characteristics of ripe fruit [11,12].

In Sicily, for centuries, the carob pod has been used in the food field, in particular in homemade pastry, due to the high sugar content (>50%) [13]. In fact, the primary seedless pod products are flour and syrup, which can be used as a chocolate or cocoa substitute. The seed exclusion from the industrial productivity is due to the differences of fat and carbohydrate composition between carob pulp and seeds, which modifies the sweet taste of the carob flour and syrup. Furthermore, these differences are shown also in terms of antioxidant activity [3]. The main marketed product carob seed based is the locust bean gum (LBG), a natural hydrocolloid, widely used in food industry as stabilizer and thickener [14]. Therefore, in the pharmaceutical field the seeds remain as a “food by-product”, a technical term to define a “food waste”.

Nowadays, there has been considerable attention toward the recovery of the waste plant matrices as possible sources of functional compounds with health properties [15]. Therefore, the purpose of the present work is to evaluate the health properties of carob seed extract and to study the effects of the ripening process on the chemical composition of the extract. In particular, we evaluated unripe (CAR-UR) and ripe (CAR-R) carob seed extracts to understand how the phytochemical composition changed during ripening process. In order to propose a suitable valorization of this food industry by-product an ultrasound-assisted extraction (UAE) technique has been used. This technique is considered an environmentally friendly extraction process, due to the use of generally recognized as safe (GRAS) solvents and the reduction of processing and residence times. Therefore, it improves the quality of bioactive product by providing higher recovery yields compared to classical extractions, and preserving also target activities of the extracts.

## 2. Materials and Methods

### 2.1. Chemical Reagents

Ethanol, 2,2’-Azobis(2-methylpropionamidine) dihydrochloride (AAPH), fluorescein (FL), 6-Hydroxy-2,5,7,8-tetramethylchroman-2-carboxylic acid (Trolox), aminoguanidine carbonate (AMG), bovine serum albumin (BSA), d-fructose, sodium nitroprusside and Griess reagent (1% dihydrochloride sulphanylamide and 0.1% naphthylethylenediamine (NED) dihydrochloride in 5% of hydrochloric acid), sodium azide, curcumin and analytical standards were purchased from Sigma-Aldrich srl (Milan, Italy). Water LC-MS Grade LiChrosolv and formic acid were obtained from Merck (Milan, Italy), acetonitrile gradient grade for high-performance liquid chromatography (HPLC) HiPerSolv Chromanorm from VWR Chemicals (Milan, Italy).

### 2.2. Plant Materials

Carob pods of *Ceratonia siliqua* L. were collected during June–August in the eastern parts of Sicily (Catania, Italy), where they naturally grow, and were identified by Professor C. Puglia, University of Catania (Italy). Samples were stored in freezer to stop ripening and analyzed within one month of collection. Seeds (10 g) were separated from the unripe (CAR-UR; **UR**) and ripe (CAR-R; **R**) fruits and then dried at different times (24 h, 48 h and 72 h; **1**, **2** and **3**) at 50 °C. Finally, they were crushed in a mechanical mill, and the resulting powder was extracted as described below.

### 2.3. Ultrasound-Assisted Extraction (UAE) Technique

The powder samples of carob seeds were extracted with a mixture of ethanol/water (40:60, *v/v*) using an ultrasonic processor (UP 400 S, Dr. Hielscher GmbH, Stuttgart, Germany) for 20 min [16]. The temperature of the extraction process was stabilized by using an ice-bath. Subsequently, the extracts were stirred at room temperature for 3 h (1st extraction; **x**) and settled overnight. The supernatant was collected, filtered through a paper filter, concentrated under vacuum to remove ethanol and lyophilized (Lio 5P-Pascal SRL, Milan, Italy).

In order to maximize the extraction process, the residual precipitate was further extracted with the hydro-alcoholic solution for 6 h under magnetic stirring and settled overnight (2nd extraction; **y**). Finally, the obtained supernatant was subjected to the same experimental procedure and the plant matrix was thrown away.

### 2.4. Analytical Determination

A total of 1 mg of the seed extracts of carob unripe (CAR-UR) and ripe (CAR-R) were treated with 600 μL of water/MeOH (5:1, *v/v*) solution. The resulting mixtures were centrifuged at 3500× *g* for 5 min. The supernatants were analyzed by HPLC-UV and liquid chromatography/mass spectrometry (LC/MS), for quantitative and qualitative determination of phenolic compounds, respectively. Dionex HPLC system (P680 pump, ASI-100 autosampler, UVD170U detector, Dionex, Milan, Italy) and Phenomenex Luna 5 μm C18 reverse-phase column (250 × 4.6 mm), thermostated at 35 °C, were used. The mobile phase was composed of 0.1% formic acid in CH_3_CN (A) and 0.1% formic acid in water (B). The elution program was: 5% A for 5 min, from 5% A to 15% over 10 min, 15% A for 5 min, 15% A to 20% over 5 min, 20% A to 25% over 10 min, 25% A to 35% over 10 min, 35% A to 50% over 10 min, flow 0.5 mL/min. Chromatograms were recorded at λ = 370 nm and 280 nm, and peak areas were used to determinate the amounts of phenolic compounds. The quantities, expressed in μg per mg of extract, were derived by standard calibration curve of following compounds: gallic acid (R^2^ = 0.997), quercetin 3-O-glucoside (R^2^ = 0.9995); quercetin 3-O-ramnoside (R^2^ = 0.997), catechin (R^2^ = 0.9993). For identified polyphenols for which standard substances were not available, the quantities were calculated in comparison with available standards containing the same aglycon [17] or similar structure. All analyses were performed at least three times, and the results were expressed as a mean ± standard deviation.

LC/MS analysis to identify the main phenolic compounds was performed by UltiMate 3000 UHPLC instrument (Thermo Fisher Scientific, Milan, Italy), equipped with Thermo Scientific Exactive Plus Orbitrap MS (Thermo Fisher Scientific) and using a heated electrospray ionization (HESI II) interface (Thermo Fisher Scientific). The column and elution program were the same of HPLC analysis. ESI-mass spectra were recorded in negative ion mode under the following conditions: heater 300 °C; capillary temperature 300 °C; nebulizer gas (nitrogen) with a flow rate of 60 arbitrary units; source voltage −3.5 kV; capillary voltage −82.5 V; tube lens voltage −150 V. Data acquisition and analysis were performed using the “Xcalibur software (Version 3.0, Thermo Fisher Scientific, Milan, Italy)

### 2.5. In Vitro Studies

#### 2.5.1. Antiglycation Activity

According to the method of Derbré et al. [18] with slight modifications, we evaluated the inhibition of fluorescence produced by AGE formation through Maillard reaction. Briefly, as optimum AGE formation, the protein model bovine serum albumin (BSA) (10 mg/mL) was incubated with d-fructose (0.5 M) in phosphate buffer 50 mM pH 7.4 (NaN_3_ 0.02%) to obtain positive controls. Native BSA sample was the negative control corresponding to no fluorescence AGE formation. The aminoguanidine (AMG) (400 μg/mL) was used as reference compounds for its AGE inhibition property [19]. The final glycated BSA solutions (300 μL) alone or in presence of samples (400 μg/mL) were incubated at 37 °C in a 96-well microtiter closed with their silicon lids for 7 days. The AGE fluorescence measurement (λ_exc_ 370 nm; λ_em_ 440 nm) is performed using a VICTOR Wallac 1420 Multilabel Counter fluorimeter (PerkinElmer, Waltham, MA, USA). The results are reported in relative fluorescence units (RFU), and the percentage of inhibition with respect to the positive control (BSA with fructose) is calculated from the following Equation (1):

*% of inhibition =* [1 − (*RFU sample/RFU − positive control*)] × 100 (1)

#### 2.5.2. Oxygen Radical Absorbance Capacity (ORAC) Assay

According to the method reported by Cao et al. [20,21,22], the antioxidant activity of CAR-UR and CAR-R was evaluated in vitro using the oxygen radical absorbance capacity (ORAC) assay. This assay evaluates the fluorescence reduction of a fluorescent probe (fluorescein solution FL, 12 nM) due to the action of peroxyl radicals generated by thermal decomposition of 2,2’-azobis(2-methylpropionamidine) dihydrochloride (AAPH, 100 mM). In the presence of an antioxidant, peroxyl radicals are scavenged and the decay of the fluorescence curve is retarded. The assay was carried out at pH 7.0 and at 37 °C, using a VICTOR Wallac 1420 Multilabel Counters fluorimeter (Perkin Elmer, Boston, MA, USA) with a fluorescence filter (excitation 540 nm, emission 570 nm). Trolox (12.5 µM) was used as the control and phosphate buffer (pH 7.0) as the blank. CAR-UR and CAR-R (1 mg/mL), were solubilized in ethanol, phosphate buffer or in their mix (50:50, *v/v*).

After thermostating for 30 min at 37 °C, AAPH was added and fluorescence measurement was begun. The fluorescence was recorded every 2 min. All measurements were expressed in relation to the initial reading, analyzing all samples, one blank and one standard at the same time. Each measure was carried out in triplicate. The ORAC value refers to the net protection area under the quenching curve of FL in the presence of an antioxidant. The results (ORAC values) were obtained and were expressed using Trolox equivalents (TE) for mg of sample (TE/mg) according to Equation (2):

*ORAC units* (*TE/mg*) = *K* (*S_sample_ − S_blank_*)/(*S_Trolox_ − S_blank_*) (2)

where *K* is a sample dilution factor and *S* is the area under the fluorescence decay curve of the sample, Trolox, or blank calculated with Origin^®^7 (OriginLab Corporation, Northampton, MA, USA).

#### 2.5.3. NO Scavenger Assay

The ability of CAR-UR and CAR-R (1 mg/mL), to inhibit the spontaneous NO production from an aqueous solution of sodium nitroprusside (20 mM) at 25 °C for 3 h was evaluated using a Griess reagent [23,24]. Curcumin (100 µg/mL) was used as reference compound, as it is a well-known molecule endowed with antioxidant activity [25,26,27]. Absorbance was measured at 540 nm with a spectrophotometer (Multiskan^®^ EX, Thermo Scientific, Waltham, MA, USA). The percent of inhibition of NO radical production was calculated according to the following Equation (3):

*% of inhibition of NO =* [*A*_0_ − *A*_1_]/*A*_0_ × 100 (3)

where *A*_0_ is the absorbance of untreated sample, *A*_1_ is the absorbance of treated samples (carob seeds).

## 3. Results and Discussion

### 3.1. Ultrasound-Assisted Extraction (UAE)

The UAE technique has been chosen for the extraction of the bioactive compounds from carob seeds. This green extraction process has been developed in order to overcome problems encountered when using conventional methods [28,29]. In fact, UAE is considered environmentally friendly, as it makes it possible to shorten processing times, to improve the product quality, to reduce the solvent amounts and, finally, to use GRAS solvents [30,31]. In addition, it is suitable for the extraction of bioactive compounds, as it provides higher recovery yields than conventional extractions and preserves the target activities of the extracts [32,33].

In order to preserve the activity of the bioactive compounds, the extraction was performed at 50 °C for 20 min, since it has been shown that longer extraction times lead to higher extraction yields [34].

Concerning the solvents, an ethanol: water mixture (40:60 *v/v*) was chosen, since it is more suitable for the ultrasound extraction of phenolic compounds [17,35].

### 3.2. Qualitative and Quantitative Analyses of Bioactive Compounds from CAR-UR and CAR-R Extracts

The qualitative analyses of principal bioactive polyphenols from carob extract were performed by LC-MS, while quantitative analyses were carried out by HPLC-UV.

In Table 1, the characteristic of distinguished compounds from CAR-UR and CAR-R extracts and corresponding tentative identities are reported. During the identification of polyphenols contained in the carob seed extracts, four different groups were distinguished: flavonols (quercetins and kaempferol derivatives), flavanols (catechins and procyanidins), gallic acid and derivatives, hydrolysable tannins. We targeted the compounds belonging to these classes, because they are biologically active natural compounds responsible for activities, impacting human health.

#### 3.2.1. Flavonols

**Figure 1:**
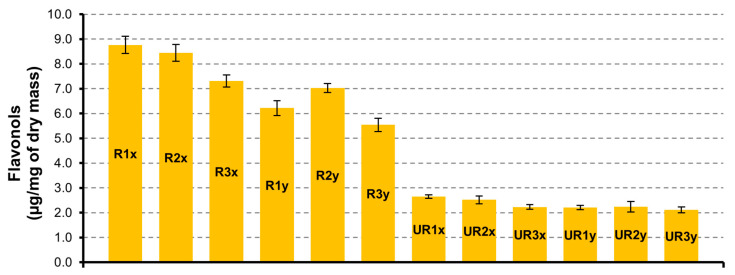
Flavonols quantification in CAR-R and CAR-UR extracts; 1 (24 h), 2 (48 h), and 3 (72 h) time of drying; x (3 h) and y (6 h) time of extraction.

In a sample from CAR-R extract, the quantity of flavonols is greater than CAR-UR extract (Figure 1). As regards to the drying time (24 h, 48 h and 72 h) of the matrix and the time of extraction (3 h and 6 h), the major quantity of biocomponents is present in the samples from carob seeds dried at 24 h and extracted for 3 h (R1x with 8.8 ± 0.3 µg/mg and UR1x with 2.7 ± 0.1 µg/mg). Among the different identified flavonols, quercetin-deoxyhexoxide is the most abundant in all series, reaching 5.2 ± 0.1 µg/mg in the R1x sample.

#### 3.2.2. Flavanols

##### Catechins

**Figure 2:**
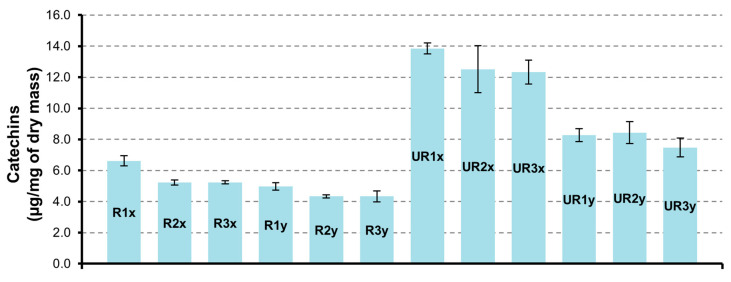
Catechins quantification in CAR-R and CAR-UR extracts; 1 (24 h), 2 (48 h), and 3 (72 h) time of drying; x (3 h) and y (6 h) time of extraction.

Differently from flavonols, the highest content of catechin and derivatives was determined in CAR-UR extract (Figure 2). The sample UR1x obtained from matrix dried at 24 h and extracted for 3 h contains a greater quantity of catechins (13.9 ± 0.4 µg/mg). In all samples the catechin is the flavanol present in larger quantities with a value of 10.8 ± 0.3 µg/mg in UR1x sample.

##### Procyanidins

**Figure 3:**
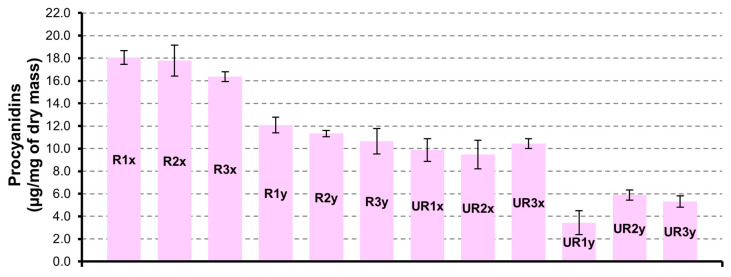
Procyanidins quantification in CAR-R and CAR-UR extracts; 1 (24 h), 2 (48 h) and 3 (72 h) time of drying; x (3 h) and y (6 h) time of extraction.

Similarly, with what was observed for flavonols, the major quantity of procyanidins (Figure 3) is found in CAR-R extracts. For both series (UR and R), the samples obtained by seed extracted for 3 h show a more abundant content of procyanidins. Unexpectedly, the UR1y sample is less rich in procyanidins compared to the UR2y and UR3y samples obtained by a drying time of 48 h and 72 h, respectively. Procyanidin dimer is the principal component of the procyanidins group, with maximum quantity of 17.4 ± 0.5 µg/mg in R1x sample.

#### 3.2.3. Gallic Acid and Derivatives

**Figure 4:**
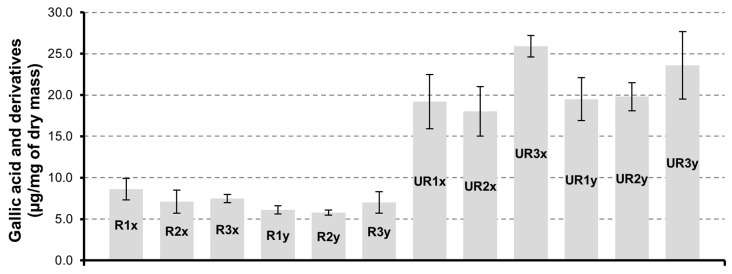
Gallic acid and derivatives quantification in CAR-R and CAR-UR extracts; 1 (24 h), 2 (48 h) and 3 (72 h) time of drying; x (3 h) and y (6 h) time of extraction.

The group of gallic acid and derivatives are more abundant in CAR-UR extracts (Figure 4). The treatment of the seed matrix under conditions of higher drying time ensures a more effective recovery of this group of compounds. In fact, the trend is in favor of products dried for 72 h and samples UR3x have the highest content of gallic acid and derivatives (25.9 ± 1.3 µg/mg). Instead, the extraction time for 3 h (x series) or 6 h (y series) does not significantly affect the gallic acid and derivatives quantities.

#### 3.2.4. Hydrolysable Gallotannins

**Figure 5:**
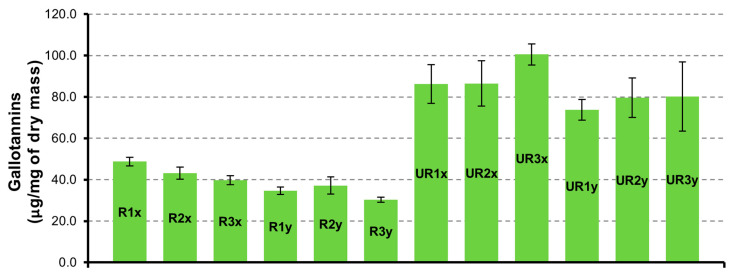
Gallotannins quantification in CAR-R and CAR-UR extracts 1 (24 h), 2 (48 h) and 3 (72 h) time of drying; x (3 h) and y (6 h) time of extraction.

The same trend observed for gallic acid and derivatives is observed for gallotannins group (Figure 5). Gallotannins are present in significant quantities in UR samples. The highest value of 100.6 ± 5.1 µg/mg is present in UR3x sample (unripe seeds, dried at 72 h and extracted for 3 h). In almost all samples, the trigalloyl-hexose is the biocomponent most abundant, reaching the value of 34.3 ± 1.1 µg/mg in UR3x sample.

### 3.3. In Vitro Studies

#### 3.3.1. Anti-Glycation Activity

Advanced glyoxidation end products (AGEs) are extensively studied as reporters of oxidative and glycooxidative damages. This condition, related to various diseases based on oxidative stress, is characterized by an increased amount of AGE contained in the tissues and body fluids, and this process, due to the formation of irreversibly fluorescent macroprotein derivatives called AGEs, occurs through the Maillard reaction, polyols pathway or lipoperoxidation. AGEs are considered reliable biomarkers of oxidative damages, and have been recognized as important pathogenetic factors of some oxidative diseases. AGEs are now considered promising drug targets, therefore researchers’ attention is focused on the development of natural/synthetic strategies capable of preventing, reducing or removing these protein oxidation products [36].

In the present work, we evaluated the inhibitory effects of CAR-R and CAR-UR extracts on the formation of fluorescent AGEs. The scientific literature, in fact, outlines the protective effect of phytochemicals, such as polyphenols, against advanced glycation of proteins [36]. From the results obtained, it seems that the effect of the extraction times (24 h, 48 h and 72 h) did not play a pivotal role in increasing the inhibitory effect of both CAR-R and CAR-UR extracts; similarly, no significant differences were found, in terms of inhibitory effect on AGE formation, between the samples obtained after the first and the second extraction. Finally, it is noteworthy that CAR-UR extract showed a considerably greater capacity of inhibition with respect to CAR-R (Table 2).

#### 3.3.2. ORAC Assay

Free radicals and reactive oxygen species (ROS) are highly reactive molecules that are generated by normal cellular processes, ROS react with cellular components, damaging DNA, carbohydrates, proteins and lipids, causing cellular and tissue injury. ORAC assay measures antioxidant activity by hydrogen atom transfer, and it provides a comprehensive analysis of antioxidant activity. Therefore, it can be used to assay the antioxidant activity of natural compounds as candidate for therapeutic use, and it has been widely accepted as a standard in vitro method to measure the antioxidant activity. ORAC assay measures both lipophilic and hydrophilic antioxidant capacity, and determines the ability of antioxidants to protect proteins from damage caused by free radicals [37,38,39]. During the ORAC assay, the decay of the fluorescence of fluorescein was monitored. The loss of fluorescence over time is due to peroxyl-radical formation by the breakdown of AAPH (2,2’-azobis-2- methyl-propanimidamide, dihydrochloride) [21]. The ORAC assay has been used to determine the antioxidant activity of CAR-UR and CAR-R. In our experiments, the difference between “area under the fluorescence decay curve” (AUC) in the presence and absence of an antioxidant was expressed into a standard Trolox calibration curve to assess the antioxidant activity as a Trolox equivalent for mg (TE/mg) of the sample.

**Figure 6:**
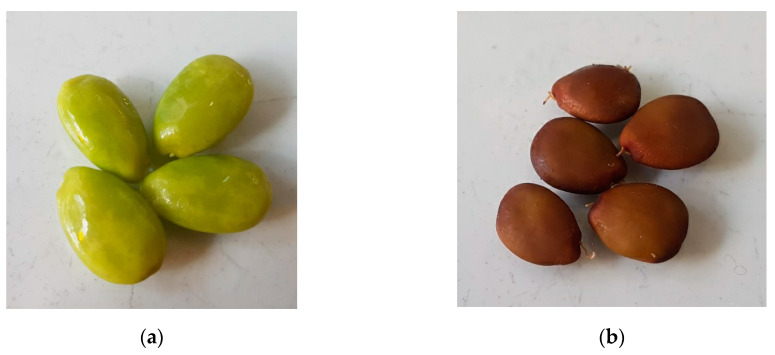
Color changes of carob seeds development. (**a**) Unripe and (**b**) ripe seeds.

As reported in Table 2, CAR-R and CAR-UR extracts showed a good ability for scavenging radicals according to the extraction time (24 h, 48 h and 72 h), while no substantial differences between the samples obtained after the first and the second extraction were observed. A noteworthy CAR-R extract showed a higher ORAC activity with respect to CAR-UR sample. This result could be explained by the analysis of characterization data in Figure 3, showing the procyanidins quantification in both CAR-R and CAR-UR extracts. As reported in Figure 3, CAR-R samples were characterized by a higher procyanidins content with respect to CAR-UR extracts, probably due to a series of biochemical reactions associated to the different degree of ripening [40]. This evidence was corroborated by the characteristics of the ripe seeds, which showed a brown coloring; a typical index of a high procyanidin content [41] (Figure 6).

Generally speaking, the fruit ripening involves a significant decrease of the phenolic compounds content, such as gallotannins, gallic acid and derivatives, catechins [42]. In the ripe carob, the decline of the phenolic content could be caused by the conversion of soluble phenolics into insoluble compounds and the oxidation of these compounds by polyphenol oxidase [43]. As reported in the literature [11], in the carob pods, the antioxidant activity decreases significantly from the unripe to ripe stage; these results are in agreement with the decline of phenolic content. Although the phenolic content decreases during ripening, our ORAC results relative the ripe extracts showed excellent antioxidant activity. This evidence is certainly attributable to the presence of procyanidins in the carob seeds, which, as previously mentioned, show a marked antioxidant activity and guarantee a remarkable sensitivity to the assay. Furthermore, these results could be attributed to the geographical origin [43,44], the Sicilian variety and extraction conditions [45]. Finally, it is well known that polymeric procyanidins have the highest antioxidant activity following quercetin, catechins and gallic acid [42,46,47]. Therefore, procyanidins show a peculiar mechanism involved in the scavenger activity, which appears more suitable to ORAC assay.

#### 3.3.3. NO Scavenger Assay

Nitric oxide is classified as a free radical, due to its unpaired electron, it exhibits remarkable reactivity with certain types of proteins and other free radicals such as ROS. Chronic exposure to the nitric oxide radical is associated with various pathological conditions, and its toxicity greatly increases when it reacts with the superoxide radical, forming the highly reactive peroxynitrite anion (ONOO−) and nitrogen dioxide (*NO_2_), which can irreversibly inhibit the mitochondrial transport chain, inducing oxidative stress and DNA mutations, and, at last, leading the cell to the apoptotic process. It has been shown that nitric oxide is directly countered by phytochemicals such as polyphenols. [48].

In Table 2, the nitric oxide inhibition capacity values for CAR-R and CAR-UR extracts are reported. The test was determined by inhibiting the spontaneous production of NO from an aqueous solution of sodium nitroprusside, as a NO donor, using Griess’s reagent [23]. CAR-UR extracts show to possess a lower percentage of NO scavenger inhibition average (27.62%) with respect to CAR-R samples showing an inhibition activity average of the nitric oxide equal to 32.3%.

## 4. Conclusions

For centuries, carob fruit has been used in the food field and the seed fraction has been considered as food waste. Nowadays, there has been considerable attention toward the recovery of the waste plant matrices as possible sources of functional compounds with health properties. Therefore, our goal was the valorization of this fraction also in relation to the modification of its chemical composition during ripening. The extraction was carried out using a friendly extraction process UAE. This innovative method was able to maximize and preserve the quality of bioactive product [31]. Seed extracts of carob unripe (CAR-UR) and ripe (CAR-R) were characterized by HPLC and LC/MS for the content of bioactive polyphenols. Finally, they were analyzed by ORAC, NO and AGEs assays, in order to estimate the antioxidant potential of the active compounds. The obtained results showed an activity strongly affected by carob degree of ripening and, consequently, by the phytochemical composition of the seeds extracts, in particular the procyanidins content.

## Figures and Tables

**Table 1:** Identification of phenolic compounds in the carob seed extracts.

Phenolic Compound	Rt (min)	UV Detection (λ (nm))	[M−H]^−^ (m/z)	Tentative Identification
1	10.2	280	331	Monogalloyl-hexose
2	13.7	280	169	Gallic acid
3	20.8	280	483	Digalloyl-hexose
4	22.2	280	577	Procyiandin dimer
5	22.6	280	865	Procyiandin trimer
6	23.6	280	289	Catechin
7	25.3	280	183	Gallic acid methyl derivative
8	26.3	280	635	Trigalloyl-hexose
9	31.8	280	787	Tetragalloyl-hexose
10	34.1	280	197	Gallic acid derivative
11	34.7	370	463	Quercetin-hexoside
12	35.2	280	441	Catechin isomer gallate
13	36.7	370	433	Quercetin-pentoside
14	37.5	370	433	Quercetin-pentoside
15	38.3	370	433	Quercetin-pentoside
16	39.2	370	447	Quercetin-deoxyhexoside
17	43.3	370	431	Kaempferol-deoxyhexoside

**Table 2:** Percentage of inhibition and antioxidant activity of CAR-R and CAR-UR extracts.

Sample	Series	Drying Time (h)	Extraction	% Inhibition AGE	ORAC UNITS	% Inhibition NO
CAR-UR	UR1x	24	1	35.32 ± 0.02	4.21 ± 0.11	30.0 ± 0.98
CAR-UR	UR1y	24	2	34.88 ± 0.53	3.43 ± 0.3	32.3 ± 0.83
CAR-UR	UR2x	48	1	34.93 ± 0.16	6.20 ± 0.6	28.3 ± 0.42
CAR-UR	UR2y	48	2	35.94 ± 0.19	7.23 ± 0.23	26.3 ± 0.63
CAR-UR	UR3x	72	1	38.17 ± 0.15	6.23 ± 0.66	23.4 ± 0.11
CAR-UR	UR3y	72	2	38.43 ± 0.22	13.28 ± 0.03	25.4 ± 0.45
CAR-R	R1x	24	1	N.D.	17.61 ± 0.76	30.7 ± 0.37
CAR-R	R1y	24	2	3.86 ± 0.86	2.75 ± 0.25	30.2 ± 0.02
CAR-R	R2x	48	1	1.88 ± 0.1	1.25 ± 0.03	32.0 ± 0.66
CAR-R	R2y	48	2	N.D.	13.47 ± 0.14	30.5 ± 0.54
CAR-R	R3x	72	1	N.D.	15.77 ± 0.54	40.7 ± 0.47
CAR-R	R3y	72	2	2.07 ± 0.2	13.96 ± 0.33	30.0 ± 0.04

ORAC Units: Trolox equivalents for µM of sample; Trolox = 1 Units ORAC; N.D. = not detectable. Data represent the mean of three independent experiments ± SD.
